# Can Iodine Revive Mercury Latent in the System?

**Published:** 1856-05

**Authors:** 


*Can Iodine revive Mercury latent in the System?*—Dr. Bache desired to call the attention of the College to the question of the influence of iodide of potassium upon workmen in mercury and lead, and upon persons who have taken medicinal preparations of those metals. It is asserted, said Dr. B., that in such cases the urine is found to contain a compound of the metal with which the system has been more or less impregnated, and that hence, when such metallic preparations have been for a long time taken, iodine must be cautiously administered. In the case of mercury, for instance, iodine is said to develop mercurial salivation with great facility. The iodine is supposed to unite with the metal deposited in the tissues, to render it soluble, and consequently to renew its activity. The facts of the case must be admitted, and the theory is plausible. It must, therefore, be presumed that when the system is thus saturated with either of the metals mentioned, but mercury in particular, the iodide of potassium ought to be very cautiously administered. In like manner, when common salt is given to persons who have taken the milder preparations of mercury, a bichloride will be formed with whatever of the metal remains in the tissues, and may produce salivation.

Dr. Jackson referred to a case which he had already mentioned to the College, and which helps to illustrate the subject under discussion. A lady, who had been taking blue pill, removed to the country, where she began to use the iodide of potassium in doses of five grains, three times a day. After taking three or four doses, she was salivated, and the medicine was suspended. When the symptoms had declined, it was resumed, and again produced the same effect.

Dr. Bell called attention to a class of cases in which the mercurial action was developed by the operation of physiological causes alone, such as cold, fatigue, &c. He had sometimes been struck with the rapid cure of syphilis where iodine was administered after mercury, and that not where full doses, such as five grains, but small and even minute quantities, were given. Yet, it was not necessary here to invoke the operation of a chemical law; the physiological operation of the iodide appeared to him sufficient to account for the results.

Dr. Page had repeatedly seen tenderness of the gums and even salivation produced by this salt, and in many of such cases no mercury had been taken, except, perhaps, in the way of a purge, but none, certainly, to impress the constitution. He doubted the proposed explanation of the ptyalism which sometimes follows the administration of iodide of potassium. This ptyalism differs from the mercurial, and notably in the circumstance of its being unaccompanied with the so-called "mercurial fetor." Besides, nitric acid will produce salivation, and perhaps other agents still. He regarded the chemical hypothesis in the case as altogether gratuitous. If lead or mercury is retained in the tissues, it must either be quiescent, or, on the administration of the iodide, be dissolved and excreted with the urine. In the one case, it is inocuous, and in the other, it passes out of the system. He believed the iodide of potassium to possess an independent and powerful physiological action, whereby it restores a healthy activity to the impaired functions.

Dr. Evans had never been able to believe that iodide of potassium developed the constitutional action of mercury. On the contrary, he knew that the salt in question was one of the best agents for checking ptyalism. Besides which, he felt sure that the iodide would of itself produce salivation. He referred to the case of a female who had never made use of any mercury, except as a cathartic; but who, on taking the iodide of potassium, became affected with a copious secretion from the Schneiderian membrane, and then from the salivary glands.

Dr. Condie had repeatedly seen tenderness of the mouth and ptyalism produced by this salt, and that without the possible intervention of mercury, as none had previously been taken. Besides, salivation by the iodide is peculiar; it produces no fetor of the breath. Salivation may likewise be caused by nitric acid, accompanied by swelling of the gums and of the salivary glands.

Dr. Jackson referred to the cases of Melsens, among which were some of paralysis cured, where the iodide of potassium developed salts of lead and potassium in the urine, although preparations of these minerals had not been taken for a long time before.

Dr. Neill called attention to the fact that metallic mercury has been found in closed cavities of the body after death. He had himself met with one case, where a considerable amount of mercury was discovered within a bronchial gland. No history of the subject of this case was attainable.

Dr. Jackson had also found metallic mercury in the brain of a man who had been accustomed to inhale the fumes of this mineral.

Dr. H. Hartshorne, passing to another subject, suggested that water, heated in copper boilers, might become poisonous. He had, in one family, observed symptoms which he could not account for, except upon this supposition.—*Transactions of the College of Physicians of Philadelphia.*

